# The environment ontology in 2016: bridging domains with increased scope, semantic density, and interoperation

**DOI:** 10.1186/s13326-016-0097-6

**Published:** 2016-09-23

**Authors:** Pier Luigi Buttigieg, Evangelos Pafilis, Suzanna E. Lewis, Mark P. Schildhauer, Ramona L. Walls, Christopher J. Mungall

**Affiliations:** 1 Alfred Wegener Institut, Helmholtz Zentrum für Polar- und Meeresforschung, Am Handelshafen 12, 27570 Bremerhaven, Germany; 2 Institute of Marine Biology Biotechnology and Aquaculture, Hellenic Centre for Marine Research, P.O Box 2214, Heraklion, 71003 Crete Greece; 3 Environmental Genomics and Systems Biology Division, Lawrence Berkeley National Laboratory, Berkeley, CA 94720 USA; 4 National Center for Ecological Analysis and Synthesis, Univ. of Calif. Santa Barbara, Santa Barbara, CA 93101 USA; 5 CyVerse, Thomas J. Keating Bioresearch Building, 1657 East Helen St, Tucson, AZ 85721 USA

**Keywords:** Environmental semantics, Habitat, Ecosystem, Ontology, Anthropogenic environment, Indoor environment, Sustainable development

## Abstract

**Background:**

The Environment Ontology (ENVO; http://www.environmentontology.org/), first described in 2013, is a resource and research target for the semantically controlled description of environmental entities. The ontology's initial aim was the representation of the biomes, environmental features, and environmental materials pertinent to genomic and microbiome-related investigations. However, the need for environmental semantics is common to a multitude of fields, and ENVO's use has steadily grown since its initial description. We have thus expanded, enhanced, and generalised the ontology to support its increasingly diverse applications.

**Methods:**

We have updated our development suite to promote expressivity, consistency, and speed: we now develop ENVO in the Web Ontology Language (OWL) and employ templating methods to accelerate class creation. We have also taken steps to better align ENVO with the Open Biological and Biomedical Ontologies (OBO) Foundry principles and interoperate with existing OBO ontologies. Further, we applied text-mining approaches to extract habitat information from the Encyclopedia of Life and automatically create experimental habitat classes within ENVO.

**Results:**

Relative to its state in 2013, ENVO's content, scope, and implementation have been enhanced and much of its existing content revised for improved semantic representation. ENVO now offers representations of habitats, environmental processes, anthropogenic environments, and entities relevant to environmental health initiatives and the global Sustainable Development Agenda for 2030. Several branches of ENVO have been used to incubate and seed new ontologies in previously unrepresented domains such as food and agronomy. The current release version of the ontology, in OWL format, is available at http://purl.obolibrary.org/obo/envo.owl.

**Conclusions:**

ENVO has been shaped into an ontology which bridges multiple domains including biomedicine, natural and anthropogenic ecology, ‘omics, and socioeconomic development. Through continued interactions with our users and partners, particularly those performing data archiving and sythesis, we anticipate that ENVO’s growth will accelerate in 2017. As always, we invite further contributions and collaboration to advance the semantic representation of the environment, ranging from geographic features and environmental materials, across habitats and ecosystems, to everyday objects in household settings.

## Background

An environment includes the natural or anthropogenic systems which can surround a living or non-living entity. This broad definition encompasses an enormous diversity of entities and scales, thus presenting numerous challenges for constructing ontologies and standards. Previously, we described the Environment Ontology (ENVO; [1]), a community-driven project which represents environmental entities including biomes, environmental features, and environmental materials. At that time, our focus was primarily on representing the environments associated with metagenomic samples: our goal was to provide a vocabulary with which to characterise sequenced environmental samples, together with an ontological structure to facilitate search, advanced querying, and inference in support of the aims of the Genomics Standards Consortium (GSC; [2]). This previous version of the ontology contained a variety of classes for describing a sample along three primary axes: the *biome* or ecosystem within which an entity of interest (usually an organism or community) is embedded; the *environmental features* that are in the vicinity of and have a strong causal influence on the entity; and the *environmental material* that is the substance surrounding or partially surrounding the entity. Although the use case is primarily microbial, the approach can encompass larger organisms – for example, a killer whale in a neritic epipelagic zone biome, present in an ecosystem defined by a marine subtidal rocky reef, and surrounded by coastal water. We also described the dynamic nature of the ontology, and the process for community extension of the ontology.

### New challenges

In the time since our initial publication, we have oriented ENVO’s development to a suite of emerging challenges extending our original and core case of describing samples of environmental and biomedical importance (e.g. [3–5]). On the one hand, sequencing projects are targeting ever more diverse environments such as city transit systems [6] and also phenomena such as soil compaction in forest ecosystems [7]. This has driven new requests from adopters such as MG-RAST [8] and the iMicrobe project (http://imicrobe.us/) which has annotated some 2813 environmental metagenomic samples with ENVO terms (see http://data.imicrobe.us/ and [9]). On the other hand, we have encountered a number of entirely new use cases in areas such as ecology and biodiversity science. Both of these fronts have, at times, required the expansion of existing branches in the ontology and, at others, required the creation of either entirely new branches, or the refactoring of existing branches. This increase in scope also presented challenges and opportunities in terms of how the ontology should be interwoven with other ontologies in the OBO Foundry and Library (http://obofoundry.org/) [10].

In this update, we describe how we have extended and in some cases broken apart ENVO to meet the above challenges. We also describe how these efforts have connected ENVO to a broader movement to further extend OBO-aligned semantics into the realm of ecology and biodiversity science [11–13], centred on co-development with ecologically themed ontologies such as the Population and Community Ontology (PCO) and Bio-collections Ontology (BCO) [14]. These efforts have been catalysed by several workshops and meetings e.g. [4] which have greatly supported ENVO in contending with entities such as habitats, environmental processes, and environmental dispositions while orienting its content to address issues of global importance.

### Expanding usage and coordination

Along with its scope, the use of ENVO is also growing and supporting data annotation, searching of datasets, and the mobilisation of sample data. For example, the journal Scientific Data (Nature Publishing Group; ISSN 2052−4463) now uses ENVO classes to annotate its Data Descriptor articles [15], allowing articles to be browsed with faceted interfaces (http://scientificdata.isa-explorer.org), and PANGAEA, a data publisher for Earth and environmental science, is continuing to use the ontology to enrich its metadata and data archives (http://www.pangaea.de). Parallel efforts such as those convened by the Global Biodiversity Information Facility (GBIF) have moved to enhance the widely used Darwin Core (DwC; http://rs.tdwg.org/dwc/; [16]) glossary by using ENVO in habitat descriptions [17]. Other users have begun to explore ENVO’s potential in data analysis [18] and in contributing to semantically aware biodiversity informatics (e.g. [19, 20]). Further, synthesis centres such as the National Centre for Ecological Analysis and Synthesis (NCEAS; Santa Barbara, USA; http://nceas.ucsb.edu/) and the Centre de synthèse et d’analyse sur la biodiversité (CESAB; Aix-en-Provence, France; http://cesab.org/) have engaged with us to explore further possibilities for usage and provide advice on coordination and community needs linked to projects such as the Data Observation Network for Earth (DataONE; www.dataone.org). Indeed, it is the diverse needs of these communities, as well as those of more recent partners (see Results and Discussion), which have compelled ENVO to develop with generalisability and versatility in mind, as is appropriate for a domain or reference ontology. Representations of microscale environments co-exist and interoperate with those of planetary-scale systems and are being further harmonised as the ontology grows in scope.

### An overview of this update

Below, we describe the updates made to improve ENVO’s ability to maintain coherence while meeting the needs of its diversifying user base and implementation partners. Our Methods section describes key technical updates while our Results focus on content-level changes. The first section of our results describes ENVO’s increased expressivity, acquired through transitioning to a more powerful development language. The second section describes the addition of processes to ENVO’s content, which has widened ENVO’s range of application and enriched the relationships between its classes. Building on its updated expressivity, the third section describes how ENVO distinguishes between environments and habitats and how thousands of habitats linked to species descriptions have been represented using text-mining approaches. Departing from the natural setting, the fourth and fifth sections describes the increased efforts made in representing anthropogenic or anthropised environments and how these changes relate to the monitoring of policy objectives and global development. Finally, we comment on how ENVO intends to handle its rapidly growing scope while maintaining expert-guided representations. From a wider perspective, we believe these updates represent multi-stakeholder convergence on the goal of integrating data through environmental contextualisation across the biosphere.

### Technical note

As a technical note, the reader is advised that OBO Library ontologies are assigned unique acronyms or initialisations, such as BFO or ENVO, that serve as shorthand identifiers for that ontology. In the following text, ontology classes (or, synonymously, ‘terms’), are written in italics and are taken from ENVO unless otherwise marked through the provision of an appropriate ontology prefix, as in ‘PATO:*laminar*’. The unique shorthand fragment of each term’s Permanent Uniform Resource Locator (PURL), e.g. ‘ENVO_00002297’ for ‘*environmental feature’*, will be included on first mention of any class, in which case the redundant namespace prefix shall be omitted. Full PURLs are of the form: http://purl.obolibrary.org/obo/ENVO_00002297, and are resolved to OWL as well as to human-readable web pages via OntoBee [21].

## Methods

The development of ENVO is now conducted using Protégé (http://protege.stanford.edu), rather than OBO Edit [22], allowing more expressivity through the Web Ontology Language (OWL). For global interoperability, we preferentially use relations from the Relations Ontology (RO; [23]) and the Basic Formal Ontology (BFO; [24]) to connect these classes. Additional relations are present, but will be incorporated into RO pending an open discussion and vetting process. The ontology is still released in both OBO and OWL formats and a number of custom exports have been made upon request (e.g. flat, character delimited formats suitable for import into relational databases, table-oriented analysis software, or network visualisation and analysis solutions). We continue to maintain obsoleted terms and link them to their replacements (where available) in a machine readable way to support automated updating of user implementations.

As with most other OBO Library ontologies, ENVO’s repository has been moved to its own GitHub “organization” (https://github.com/EnvironmentOntology). This change does not affect downstream users who consume the ontology using standard permanent URLs; however, it does provide a better mechanism for stakeholders to become involved with the development of the ontology through, for example, an improved issue tracker [25]. Further, it allows easier reference to previous versions of the ontology for backwards compatibility.

OWLTools (https://github.com/owlcollab/owltools) and ROBOT [26] (https://github.com/ontodev/robot/) are currently being used for release management, and for the import of classes from other OBO Foundry and Library ontologies in alignment with the Minimum Information to Reference an External Ontology Term (MIREOT; [27]) guidelines. These import procedures are primarily used to express environments that are dependent on entities defined outside of ENVO. For example, environments defined by anatomical entities and chemical entities are expressed using classes from ontologies such as the Uber Anatomy Ontology (UBERON; [28]) and the Chemical Entities of Biological Interest Ontology (CHEBI; [29]) to prevent duplicating existing, well-developed semantics relevant to terms such as *‘xylene contaminated soil’* [ENVO_00002146] and *‘axilla skin environment’* [ENVO_08000001].

We have created a TermGenie instance (http://envo.termgenie.org/) [30] that allows for web-based addition of new terms that conform to a pre-defined template, or following a free-form pattern. We are also documenting our design patterns (ODPs) using the emerging ‘dead simple owl design patterns’ standard (https://github.com/dosumis/dead_simple_owl_design_patterns) and are using these patterns to generate small portions of the ontology. Further, we have begun to use the results of text-mining approaches, noted in [1], discussed below, and documented by Pafilis et al. [31], to automatically generate experimental classes which, upon curation, can be integrated into the core ontology.

## Results and discussion

ENVO now includes some 2159 classes primarily representing biomes, geographic features, and environmental materials, along with 18,791 axioms (logical statements) defining, interconnecting, and interrelating them. This contrasts with 1644 classes and 14,542 axioms present when ENVO’s original description was published. The growth of the ontology was primarily driven by the needs of the ‘omics community using the Minimal Information about any (x) Sequence (MIxS; [32]) checklist and its extensions such as MIxS for the Built Environment (MIxS-BE; [33]). These needs were communicated through individual requests for new classes and requests coordinated through, for example, curation efforts of organisations such as the European Nucleotide Archive (ENA) (e.g. [34]). More currently, the bulk of the changes to ENVO’s content have been motivated by the ontology’s growing adoption and engagement with new user communities as well as the need to integrate their varying approaches to describing environments.

### Increases in semantic density and expressivity

**Fig. 1:**
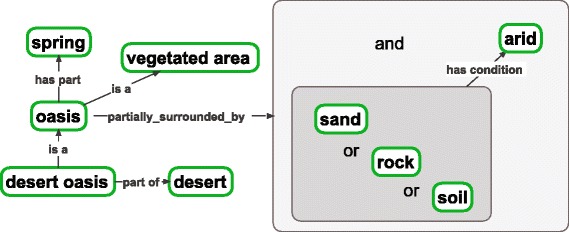
Illustrative example of ENVO’s improved semantic expression with OWL axioms. An oasis is a vegetated area which has, as a part, a spring and is surrounded by an arid portion of soil, rock, or sand

As we are now developing ENVO using the expressivity of OWL (see Methods), we have increased the variety and density of linkages between many of ENVO’s classes as well as the detail in their logical definitions. This increased semantic density offers more flexibility when using the ontology for querying, inference, and semantically enhanced analysis. To illustrate the increased expressivity, an *oasis* [ENVO_01001304] (Fig. 1) is represented as a subclass of *‘vegetated area’* [ENVO_01001305] which has, as a part, some *‘spring’* [ENVO_00000027] and is partially surrounded by a portion of either *rock* [ENVO_00001995], *sand* [ENVO_01000017], or *soil* [ENVO_00001998] which, itself, is *arid* [ENVO_01000230]. This representation has several facets which involve type hierarchy (i.e. class and subclass relationships), parthood, and adjacency, and which define key properties of one or more of the classes involved. Practically, users and machine agents can now identify an *oasis* (and any data that has been associated with that class) by any one of these routes such as querying for a *vegetated area* that is surrounded by *arid* environmental materials or which has a *spring* as a necessary part.

The increased axiomatisation described above has also improved our ability to represent semantically problematic classes such as ‘*hydrographic feature’* [ENVO_00000012] and *‘marine pelagic feature’* [ENVO_01000044]. The issues with these somewhat artificial or convenience groupings are discussed in [1]; in brief, their membership is dictated more through convention than physical or formative similarities, often adding ambiguity and confounding search and inference. For example, one is correct in asserting that a lighthouse, a lake, and a coral reef are hydrographic features due to the nautical conventions of hydrography; however, these entities are substantially different from one another and much better distributed in hierarchies true to their physical attributes and/or the processes of their formation. With ENVO’s greater semantic flexibility, the varied criteria for including a class in one of these convenience groupings can be more precisely defined and classes which satisfy these criteria can be interlinked through automated inference: the action of reasoning software which can use logical statements to infer relationships and hierarchies which were not asserted by a human. For example, any class which has been asserted to be *‘adjacent to’* some ‘*water body’* or ‘*partially surrounded by’* some ‘*water’* will be inferred to be a subclass of *‘hydrographic feature’*. Similarly, *‘marine pelagic feature’* would be populated by any entity which has been asserted to be *‘part of’* some *‘marine water body’* or *'composed primarily of'* some *‘sea water’*. Similarly, many subclasses of *‘environmental material’* [ENVO_00010483] are now placed in inferred hierarchies using various subclasses of *quality* [PATO_0000001] such as *‘quality of a solid’* [PATO_0001546], *‘quality of a gas’* [PATO_0001547], and *‘quality of a liquid’* [PATO_0001548]. Such assertions provide a way to construct and populate classes like “solid” or “liquid” through inference, avoiding asserted multiple inheritance while simultaneously preserving clear representations based on multiple criteria.

As illustrated above, the flexibility that comes with increased axiomatisation is an important step in supporting multiple, varying classifications of environmental entities in an integrated fashion. We will leverage these capacities to disentangle the semantics of environmental entities across user groups which use different definitions for syntactically similar terms. The hundreds of official and operational definitions of “forest” [35], which can influence critical decisions in conservation and sustainable land use [36, 37], will be one of our first targets in this process. We anticipate that ENVO will host multiple classes representing the different entities typically gathered under one label, using synonym lists and cross-references to relevant definition sources to untangle alternative term usage. This approach will allow a diversity of users, including those with limited exposure to semantic technology, to easily identify which class they wish to employ. Simultaneously, advanced users can take advantage of ENVO’s continually developing axiomatisation to perform analyses of such semantic spaces and to mobilise data in novel ways. In addition to axiomatisation of relatively static entities (or *continuants* [BFO_0000002]), we aim to further extend this flexibility through the representation of processes, described in the following section.

### Representing environmental processes

**Fig. 2:**
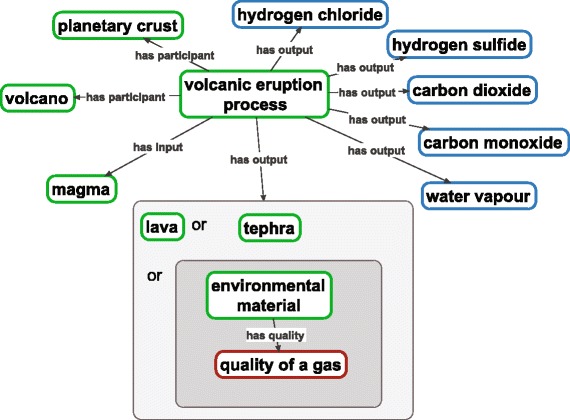
Illustrative example of ENVO’s representation of a volcanic eruption process. ENVO’s new environmental process branch allows the participants, inputs, and outputs of environmental processes to be represented. These entities may be from ENVO’s content or imported from other ontologies, promoting interoperation and orthogonality. *Green* borders indicate ENVO classes, *blue* borders CHEBI classes, and *red* borders PATO classes

The material parts of environments are constantly changing and the representation of the processes which are involved in such changes naturally falls in ENVO’s scope. Consequently, an initial set of some 53 classes representing environmental processes, aligned with the *process* [BFO_0000015] class in the Basic Formal Ontology, have been added to ENVO and have been used to interlink material entities throughout the ontology. As an example, a ‘*volcanic eruption’* [ENVO_01000634] ties together *magma* [ENVO_01000648], *lava* [ENVO:01000231], *tephra* [ENVO:01000660], and a set of gaseous materials through relations of input, output, and more general participation (Fig. 2). Inference (described above) can be used to populate processes such as *‘carbon-bearing gas emission process’* [ENVO_01000742] with both natural and anthropogenic processes based on their inputs and outputs. Such constructions can be used to efficiently represent higher-order processes such as *‘climate change’* [ENVO_01000629]. The relations between these classes are primarily controlled by the Relations Ontology (RO, [23]) and work is underway to update both ENVO and RO to offer more powerful expression.

Further, processes can be used to define entities which arise as a result of their instantiation in a more machine-actionable manner. For example, an *‘igneous intrusion process’* [ENVO_01000657] may be linked to an *‘igneous intrusion’* [ENVO_01000659] through RO’s *‘formed as a result of”* [RO_0002354] relation. Historically, many of the RO relations connecting processes with independent continuants have primarily been applied to the biological process hierarchy of the Gene Ontology [38], and may require some generalisation for environmental processes. We hope to expose these as ENVO’s process hierarchy develops and lead the extension of relational semantics into ecological and environmental domains.

In addition to their immediate utility, classes representing environmental processes allow a key point of interaction with other ontologies and semantic resources. To illustrate, participation in a *‘land consumption process’* [ENVO_01000743] may encompass material and immaterial anthropogenic and natural entities such as: buildings; legal documents and rights; indigenous populations and lands; and ecosystems. This will be essential to interweave ontologies across broad “super-domains” such as sustainable development (see Environmental Semantics in support of the Sustainable Development Agenda for 2030, below) as well as articulate threats to habitats.

### Clarifying and representing habitats

Interest in a given environmental system and the processes which change it is often driven by the desire to understand the ecology of the organisms that inhabit it. The relationship between populations of organisms and the one or more environmental systems needed to sustain their existence and growth is the foundation for the semantics of “habitat”. ENVO’s previous representation of habitats was underdeveloped, and many of its classes confounded the semantics of “environment” and “habitat”, primarily due to the loose usage of these terms across disciplines. Thus, as anticipated by Buttigieg et al. [1], ENVO’s semantically confounded *habitat* [ENVO_00002036] class has been made obsolete and replaced by the equivalently labelled, *habitat* [ENVO_01000739]. ENVO’s current *habitat* class represents an environmental system within which an ecological population (i.e. *population* [PCO_0000001]), can persist and grow. Importantly, a *population* of a given species (or similar grouping) need not be present in such an environmental system in order for that system to qualify as that species’ habitat: the environmental system need only have the disposition to support such a population.

**Fig. 3:**
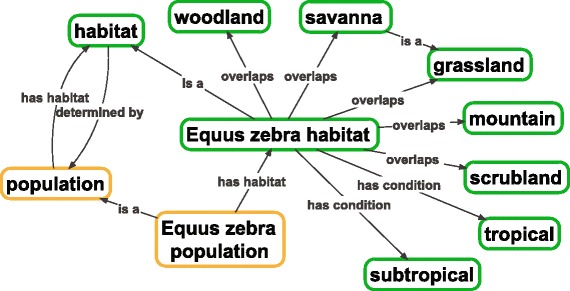
Typical formulation of an experimental habitat class in the ENVO’s automatically generated habitat content. Here, the environmental system which can support the survival and growth of a population of *Equus zebra* is represented. Based on the Encyclopedia of Life’s descriptions of the environments in which such populations are typically found, their habitat is represented as an environmental system which overlaps these environments. The boundaries of the habitat are determined by the physiological tolerances of the organisms: the habitat ends where the potential for an organism (or mating pair of organisms) to survive and/or increase their population size is no more. See text for comments on handling different definitions of ‘habitat’. *Green* borders indicate ENVO classes, while *orange* borders represent PCO classes

Typically, subclasses of the current *habitat* will be formulated similarly to ‘*Equus zebra habitat’*, in that they will always reference some species or other grouping of organisms with similar physiological tolerances and environmental preferences. Habitats can be related to their constituent environments using the *overlaps* [RO_0002131] relation, as any given habitat will share parts with a range of environment types, according to the requirements of the species of interest. Organisms and populations of organisms can be associated with their habitats by the *‘has habitat’* [RO_0002303] relation, the definition of which was updated as a result of ENVO’s clarified representation of *habitat*. See Fig. 3 for illustration of these semantics.

As most collections of organisms grouped at species (and even sub-species) level can be associated with a distinct habitat, the number of classes in this branch is likely to become very large and automated approaches are required to make reasonable progress. Thus, we created an experimental branch of ENVO based on the results of the ENVIRONMENTS-EOL project [31], which text-mined the habitat descriptions of the Encyclopedia of Life [39] and associated them with ENVO classes. This approach generated results for 227,583 taxa, associating them with 1,605,974 automatically generated annotations (“tags”) based on ENVO class labels and synonyms. We reduced this collection to 112,585 taxa by removing taxa which we were unable to link to a National Center for Biotechnology Information (NCBI) taxonomy entry via the EOL API. This filtering was performed to focus on taxa that we could readily map to a widely-used taxonomy which is integrated with genomic data. We acknowledge that other taxonomies and/or phylogenies may be more accurate, both globally and for specific taxa: initiatives such as the Tree of Life Web Project [40], PhylomeDB [41], and TreeBase [42, 43] are of great interest in enhancing this dimension of our habitat hierarchy, and we will work towards integrating additional taxonomic resources in future releases. We then chose to focus our attention on taxa which face threats to their persistence by retaining only those taxa which feature in the International Union for Conservation of Nature (IUCN) Red List [44] as extinct in the wild (EW), regionally extinct (RE), critically endangered (CR), or endangered (EN), yielding 5,117 taxa. Due to their experimental nature, the results of this exercise are stored in a separate repository [45] and classes exist in a temporary ID range (prefixed with “ENVO:H”). The complete result set may be retrieved from [46].

Our automatic mapping provides a foundation upon which high-quality semantic resources can be created for linking organisms to the environments which sustain their populations. However, this automatic mapping is prone to error and must be refined. Erroneous mappings have been identified due to simple false positives, ambiguous class labels, and text-mining routines which only account for the basic structure of the ontology. False positives can easily arise from the parsing of place names such as “Mountain River” or from other largely unpredictable facets of natural language. An example of the latter two issues was apparent in the overly narrow association of class label *‘pelagic zone’* [ENVO_00000208] to marine ecosystems. Large lakes are also said to have pelagic zones, however, workers in both marine and lacustrine domains will generally omit labels with qualifiers such as “*marine* pelagic zone”. Within ENVO, we decided to err on the side of caution and employ such modifiers, maintaining “pelagic zone” as a broad synonym associated with each class. Enhanced text-mining techniques, such as natural language processing (NLP), statistical analysis of text-mining results, and additional filtering based on a term’s ontological context, could further reduce false positives. We have yet to explore the feasibility of this solution with a rapidly developing ontology like ENVO, but we are encouraged by the promise of semi-automated ontology growth in the environmental domain.

While ENVO’s preliminary habitat representation shows promise, we stress that refinement and curation are needed before habitat classes will be added to the release version of the ontology. We will solicit input from experts on particular species and their environmental preferences in order to validate our mapping, report poor representations, and request enhancements via the ENVO issue tracker [25]. Building on these initial results, we aim to enable semantically controlled, large-scale habitat analyses driven by text-mining as described by authors such as Groom [47]. Eventually, we anticipate that coupling habitat semantics with distributional e.g. [48], trait e.g. [19], or behavioural data will offer further opportunities in predicting multi-scale patterns of biodiversity.

Importantly, it must be acknowledged that there will be some ambiguity in what constitutes an ecological population and, hence, what environments can provide a *habitat* for its members. Further, definitions of “habitat” also vary (see, e.g. [49, 50]), increasing the need for structured representation of the semantics behind the entity. These issues are further complicated by the decoupling of phylogeny from function due to, for example, horizontal gene transfer in microbes as well as procedural issues in stably identifying units of diversity [51–53] along with the role of microdiversity [54, 55]. Definitional variation and ambiguous boundary conditions are not unusual in the representation of environmental entities. ENVO will remain agnostic regarding any definition’s ‘correctness’ and we anticipate that co-existing and semantically overlapping habitat classes will emerge to represent the entities referenced by different communities. Addressing this challenge will be greatly helped by ENVO’s increased semantic flexibility, described in the sections above, which will be leveraged to tease apart this space. Through this process of representation, we hope that ENVO will serve as a hub for healthy and structured debate over central ecological entities such as habitats and niches, while simultaneously providing a resource to mobilise data in transparent ways. As a final but important note, we frequently encountered information indicating the typically deleterious impact of human activity on habitats. This, along with the need to provide semantics for defining anthropised environments, has motivated updates in ENVO’s representation of human-centric environmental systems and processes, as we describe below.

### Anthropogenic environments and impacts

Much of ENVO’s recent development has been guided by requests for the representation of anthropogenic environments. This is bolstered by the clear need to provide semantics for the interplay of human activity with natural systems, echoing Ellis and Ramankutty’s call for ecologists to increase their focus on anthropised environments which now dominate the Anthropocene Earth [56]. To illustrate, requests linked to the Program for Resistance, Immunology, Surveillance and Modeling of Malaria in Uganda (PRISM), a project in the framework of the International Centers of Excellence for Malaria Research (ICEMRs; see e.g. [57] for context), have motivated the creation of classes representing housing materials, building components, and building types relevant to the assessment of malaria risk [58]. Examples include *concrete* [ENVO_01000458], *'sheet-iron building roof'* [ENVO_01000510], and ‘*ventilated improved pit latrine’* [ENVO_01000530]. With similar motivation, classes of *vehicle* [ENVO_01000604] and classes for mesoscopic objects such as *lamp* [ENVO_01000566] and *lantern* [ENVO_01000565] have also been added.

Additions motivated by PRISM demonstrate how ENVO’s content has been shaped by the needs of an environmental health initiative. We plan to link such efforts to the representation of pathogen or vector habitats to draw together knowledge on the build environment and pathogen ecology. Methods such as DISEASES [59] and Bio-Lark [60] are particularly promising in this regard, leveraging text-mining to discover and link terms from the medical and biological domains. ENVO classes such as *slum* [ENVO_01000653] and *factory* [ENVO_01000536] can complement these use cases and reinforce the representations of anthropogenic environments. This work will also produce content which addresses the needs of projects investigating the microbiomes of indoor environments [61–65]. Classes representing building parts such as *‘living room’* [ENVO_01000423], *patio* [ENVO_01000424], and *‘indoor kitchen’* [ENVO_01000421] are being used in the annotation of metagenomes [34] and exemplify a convergence of needs which provide a foundation for broad interoperability through environmental semantics.

In parallel to object-type classes, ENVO’s material hierarchy is being populated with anthropogenic materials. The ontology has been identified as a means to support the assessment of nanomaterial risk in environmental systems [66] and has classes which are immediately useful. For example, classes such as *‘carbon nanotube enriched soil’* [ENVO_01000427] combine *soil* [ENVO_00001998] and *‘carbon nanotube’* [CHEBI_50594] in a pattern which is easily propagated. Materials associated with health concerns, such as *‘fine respirable suspended particulate matter’* [ENVO_01000415] (i.e. PM 2.5), have been added and will be integral to ENVO’s role in environmental monitoring efforts (see below). Our aim is to provide semantics capable of supporting the clarification and interoperability of information used to assess human impacts on ecosystems e.g. [67], while promoting collaboration between environmental and material researchers and refining ENVO’s content.

Taken with ENVO’s increased coverage of natural environments, these updates have prepared the ontology to address challenges in planetary monitoring across scales. We have begun to realise this potential through engagement with the Sustainable Development Agenda for 2030, summarised below.

### Environmental semantics in support of the Sustainable Development Agenda for 2030

Over the course of 2015 and in collaboration with the United Nations Environment Programme (UNEP), the Sustainable Development Goal Interface Ontology (SDGIO; [68]) has been founded with the aim to provide a semantic resource for the Sustainable Development Goals (SDGs, [69–71]), their targets, and indicators. Environmental semantics strongly feature in this effort and ENVO – a key component of SDGIO – is being shaped by its demands. ENVO’s increased axiomatisation, process semantics, and representation of anthropogenic environments (described above) will be brought to bear to represent the entities associated with terms across multiple SDG-linked official vocabularies such as the General Multilingual Environmental Thesaurus (GEMET; [72]). To illustrate, we have created processual classes expressing environmental hazards and disasters such as *earthquake* [ENVO_01000677], *tsunami* [ENVO_01000689], ‘*volcanic eruption’* [ENVO_01000634], and *drought* [ENVO_1000745] as well as continuant classes such as ‘*atmospheric water vapour*’ [ENVO_01000268], linked to roles such as ‘*greenhouse gas*’ [CHEBI_76413], to support the handling of information for Target 13.1 (“Strengthen resilience and adaptive capacity to climate-related hazards and natural disasters in all countries”) of SDG 13 (“Take urgent action to combat climate change and its impacts”). These new categories will be supported by leveraging ENVO’s representation of environmental conditions to address the semantics of weather and climate. Further, we have introduced classes representing forest types (relevant to Targets in SDG 15, Targets 6.6), which are being aligned to definitions in the Global Forest Map (GFM) 2000 [73]. Anthropogenic and anthropised environmental entities as well as axioms tying together continuants and processes, discussed above, will play an especially important role in addressing many of the SDGs. We will continue to add content to support the SDGs in all of ENVO’s branches and invite the wider community to participate via our issue tracker [25].

The enhancements above have set the foundation for interlinking data described with ENVO to global policy targets through SDGIO via constructs such as ecosystem functions and services. We aim to develop this capacity and facilitate the exposure of scientific outputs to the policy community as they become increasingly driven by data products. Early work exploring this potential is underway and aims to semantically annotate and expose outputs of the Frontiers in Arctic Marine Monitoring (FRAM; http://fram-data.awi.de/) programme [74] with ENVO and SDGIO, linking data about fragile Arctic ecosystems to SDGs 13 and 14 (“Conserve and sustainably use the oceans, seas and marine resources for sustainable development”). We encourage other projects, be they single investigations or observatory-scale endeavours, to contact us should they wish to coordinate similar efforts.

### Handling an ever-growing scope

“The environment is everything which isn’t me” – Albert Einstein.

It is readily apparent that the range of entities represented in ENVO is expanding very rapidly, well beyond its original objectives within the context of the GSC. In many cases, this expansion is due to a lack of similar resources in domains such as architecture, development, or food and agriculture. As environmental systems can feature an immense range of components, it is valid to extend ENVO’s content to address these domains. However, it far more desirable that such entities are represented in independent, expert-led ontologies restricted to a disciplinary domain in order to preserve semantic orthogonality and improve accuracy [75]. This would by no means diminish ENVO’s capacities or content: ENVO can readily import classes from such domain ontologies to represent the environments they are components of and preserve its current scope in a more sustainable way. Indeed, this strategy has been successfully applied to ontologies such as the metazoan anatomy ontology, UBERON, which federates with separate ontologies dedicated to non-chordate clades, such as sponges, ctenophores and cephalopods (see Methods).

Driven by the rationale described above, we have recently begun to use ENVO’s content to seed new domain ontologies. For example, we are contributing to the launch of a food ontology (FOODON; [76]), to which we have transferred ENVO’s ‘*food product’* [ENVO_00002002] classes including *amasake* [ENVO_00003872], *‘bambara groundnut product’* [ENVO_0010109], and *‘zebra milk’* [ENVO_02000018]. Further, we are co-developing agronomy and agriculture related semantics with the newly launched Agronomy Ontology (AgrO; [77]), led by members of the CGIAR (http://www.cgiar.org/) and Bioversity International (http://www.bioversityinternational.org/). As noted above, we are also in the process of launching an application ontology for polar oceanographic, biogeochemical, and biological observation linked to the FRAM programme by enhancing ENVO’s content for polar investigations [74]. ENVO’s role in the growth of the SDGIO will very likely produce more targets for this approach, such as urban infrastructure systems and disaster response systems. In summary, ENVO is likely to handle its ever-growing and highly diverse content by serving as an incubator for the ontologies of orphan domains, aligning them with the best practices of the OBO Foundry and promoting their interoperation with existing resources. We welcome adopters of these proto-ontologies and offer assistance in launching new ontologies to sustainably extend an ever more comprehensive semantic layer.

## Conclusion and outlook

The growing interest in and use of ENVO has motivated notable expansion and enhancement of the ontology, while simultaneously creating new challenges. The addition of environmental processes and dispositions has extended ENVO’s semantic range and supported our efforts to increase its axiomatic density. We have made progress in representing thousands of habitats using methods driven by text-mining and look forward to refining this content to catalyse efforts to synthesise ecological data with clear semantic representation. Furthermore, we have begun to align ENVO with key themes in global conservation and development. Future efforts will concentrate on the representation of entities described by initiatives such as the IUCN Red List of Ecosystems [78] and relevant to the Sustainable Development Agenda for 2030, demanding a cohesive treatment of environments with varying degrees of human impact. We see these efforts as a contribution towards e-infrastructures able to address the grand challenges of sustainably managing Earth’s ecosystems, as articulated by Hardisty et al. [79] and in line with rationale of the Bouchout Declaration’s aims of creating open standards for integration and sharing of (http://www.bouchoutdeclaration.org/declaration/) along with the recently released FAIR principles [80].

We anticipate that the path ahead will require greater technical enhancements, contributions from the communities it supports, and a broader team of developers in order to facilitate and expedite its development alongside that of the nascent ontologies nested within ENVO’s hierarchies. We have begun to employ tools such as TermGenie [30] and ROBOT [26] to address these needs. Further semantic diversity, leveraging Basic Formal Ontology 2.0 (BFO) classes such as *history* [BFO_0000182], will be explored to formulate classes representing ecological succession and paleoenvironmental entities. Additionally, we plan greater interaction with initiatives such as GloBI [20] to improve the representation of organismal interactions in environmental systems and to make ENVO’s semantics more relevant to archetypal ecological data sets. Furthermore, we hope to expand our collaboration with synthesis centres and data integrators and are exploring new possibilities with, for example, the Integrated Digitized Biocollections (iDigBio) National Resource for Advancing Digitization of Biodiversity Collections (ADBC) [81].

As always, we extend an invitation to communities of ontologists, informaticians, domain experts, and other current or new users of the semantic layer to interact with and shape ENVO to their needs. We especially welcome groups wishing to adopt the nascent domain ontologies forming within ENVO and users who are able to test whether ENVO’s logical structure can enhance their data analyses. We look forward to broadening and deepening the semantic layer for the environmental sciences.

### Downloads

ENVO’s latest release version is available for download [82]. A file including only ENVO classes (envo-basic.obo) is available as well as files with additional classes from ontologies used to construct logical definitions in ENVO (envo.obo and envo.owl). The ontology is available both in OBO and OWL format; however, the OWL format features greater expressivity and is a W3C recommendation for semantic representation of objects on the Web (www.w3.org/2004/OWL/). The OBO Library page for ENVO (http://obofoundry.org/ontology/envo.html) also contains an index of available downloads plus links to various browsers offering ENVO. As before, this ontology is free and open to all users and is distributed under a CC-BY license.
